# Associations between physical activity, physical fitness, sedentary behavior, and mathematics achievement in school-aged children: a cross-sectional study

**DOI:** 10.3389/fcogn.2026.1903208

**Published:** 2026-09-16

**Authors:** Souhail Hermassi, Ayat Alshaer, Abdulla Alneama, Sascha Ketelhut, Roland van den Tillaar

**Affiliations:** 1 Physical Education Department, College of Education, Qatar University, Doha, Qatar; 2 Institute of Sport Science, University of Bern, Bern, Switzerland; 3 Department of Sport Sciences and Physical Education, Nord University, Levanger, Norway

**Keywords:** cognitive performance, exercise, motor control, sedentary behavior, sex differences

## Abstract

**Aim:**

This study examined associations between physical activity, physical fitness, and academic achievement in school-aged children, considering potential sex differences.

**Methods:**

A total of 126 schoolchildren (12.2 ± 0.4 years) participated in this cross-sectional study and were classified as physically active (*n* = 63) or non-active (*n* = 63) based on self-reported moderate-to-vigorous physical activity (MVPA) assessed using the International Physical Activity Questionnaire–Short Form (IPAQ-SF). Children reporting ≥300 min/week of MVPA were classified as active. Physical fitness was assessed using the medicine ball throw, Stork balance, and handgrip strength. Peak height velocity (PHV) was estimated using Moore's equations, and academic achievement was assessed using mathematics percentage scores. Two-way ANOVA and Pearson correlations examined group differences and associations.

**Results:**

Active children had lower body mass than non-active children (boys: 47.2 ± 12.9 vs. 53.8 ± 15.0 kg; girls: 50.6 ± 9.5 vs. 53.8 ± 15.7 kg; *F* = 4.2, *p* = 0.042, *d* = 0.37). Mathematics scores were higher in active children (boys: 90.2 ± 5.8 vs. 76.3 ± 7.9%; girls: 89.5 ± 6.2 vs. 77.1 ± 8.1%; *F* = 96.8, *p* < 0.001, *d* = 1.7). Fitness performance was generally higher in active than non-active children (*F* ≥ 3.98, *p* ≤ 0.048, *d* ≥ 0.36). Significant sex effects were observed for medicine ball throw and handgrip strength, with no significant group × sex interactions. Active boys reported the highest vigorous activity (1,209 ± 732 METs/week) and lowest sedentary time (2.6 ± 1.3 h/day). Sedentary time was moderately negatively correlated with mathematics scores (*r* = −0.48, *p* < 0.001), whereas total MET-min/week showed a trivial positive correlation (*r* = 0.18, *p* = 0.049).

**Conclusion:**

Physically active children demonstrated higher physical fitness and mathematics achievement than non-active peers. These findings support cross-sectional associations among physical activity, physical fitness, sedentary behavior, and mathematics achievement. However, because of the cross-sectional design and the absence of statistical control for potential confounding factors such as biological maturation, causal or independent relationships cannot be inferred.

## 1 Introduction

Physical inactivity and sedentary behavior among children and adolescents have become a global public health concern, with implications for both physical health and cognitive development. Recent international surveys highlight that a substantial proportion of youth fail to meet recommended activity levels, underscoring the urgency for effective interventions. Physical activity and sedentary behavior are distinct yet interrelated lifestyle patterns that together encompass all waking hours of the day. Physical activity is defined as any bodily movement produced by skeletal muscles that results in energy expenditure above resting levels, encompassing activities performed during leisure, work, or daily living (Caspersen et al., 1985; Spruit et al., 2016). Rising levels of sedentary behavior and declining physical activity among children and adolescents have become an escalating public health issue (Rodriguez-Ayllon et al., 2019; Wang et al., 2024). A growing proportion of youth fail to achieve the recommended standards of daily physical activity (Hu et al., 2024). Current international guidelines recommend that children and adolescents engage in at least 60 min of moderate-to-vigorous physical activity (MVPA) per day, in addition to limiting recreational screen time to less than 2 h per day (Casanova et al., 2023). Findings from a World Health Organization survey involving 72,845 school-aged children across 34 countries reveal that a substantial number do not participate in adequate physical activity, with only approximately 40% achieving the recommended daily MVPA, and nearly one in three leading predominantly sedentary lifestyles (Guthold et al., 2010). These trends underscore the urgent need to understand not only physical activity patterns but also how such behaviors may translate into physiological adaptations that influence learning outcomes.

Physical activity plays a vital role in promoting children's physical and mental health, contributing to improved strength, endurance, and overall physiological functioning (Bertrand et al., 2025; Casanova et al., 2023). Beyond these health benefits, accumulating evidence indicates that physical activity is positively associated with cognitive performance and academic achievement, particularly when activity is integrated into school routines (Hermassi et al., 2023a; Ketelhut et al., 2020; Martin-Martinez et al., 2023; Shah and Diwan, 2025; Teuber et al., 2024). Regular engagement in physical activity has been linked to enhanced concentration, executive function, and emotional regulation, which may support learning and academic success (Castillo-Navarrete et al., 2024; Hermassi et al., 2025; Ketelhut et al., 2024; Singh et al., 2025; Zhang et al., 2025). Importantly, physical fitness may represent a potential correlate of both physical activity and academic outcomes. However, no mediation analysis was performed in the present study, and any mediating role should therefore be considered hypothetical. However, the relationship between physical activity and academic performance is complex and may be influenced by additional factors such as physical fitness, sedentary behavior, and the type or intensity of activity (Hermassi et al., 2024a; Teuber et al., 2024; Wang et al., 2024). Understanding these relationships is critical, as many children fail to meet recommended physical activity levels, and excessive sedentary behavior is increasingly common. Despite the recognized benefits of physical activity, relatively few studies have simultaneously examined physical activity patterns, health-related physical fitness, and academic achievement in school-aged children, while considering potential sex differences. Clarifying these relationships is necessary to better understand how physical activity, physical fitness, sedentary behavior, and academic achievement are associated in school-aged children.

A key conceptual distinction that warrants clarification is the difference between physical activity (a behavior), physical fitness (a physiological attribute), and academic achievement (an educational outcome). Physical activity represents a modifiable behavioral exposure. Physical fitness—including muscular strength, power, balance, and cardiorespiratory capacity—reflects physiological adaptations that may result from habitual engagement in physical activity. Academic achievement, in contrast, is a multidetermined educational outcome influenced by cognitive ability, instructional quality, socioeconomic context, sleep, nutrition, biological maturation, and psychosocial factors. Within this conceptual model, Physical fitness has been proposed in the literature as a potential correlate of both physical activity and academic outcomes. However, the present study did not evaluate mediation pathways, and all observed relationships should be interpreted as cross-sectional associations. These constructs should therefore not be treated as interchangeable. Failure to distinguish behavioral exposure (physical activity) from physiological status (fitness) may lead to conceptual ambiguity and overinterpretation of associations. The present study explicitly differentiates these constructs in both design and interpretation.

While numerous studies have investigated cardiorespiratory fitness in relation to academic performance (Aly et al., 2024; Barbosa et al., 2020; Bielik et al., 2025; Hermassi et al., 2023a, 2024b; Kao et al., 2021; Li et al., 2023; Robinson et al., 2023), fewer have examined the contribution of muscular fitness components such as strength and power (Aly et al., 2024). A meta-analysis by Robinson et al. (2023) reported small but positive effects of strength training and muscular fitness on cognition and academic outcomes, although the certainty of the evidence was low. These findings suggest potential mechanisms linking physical fitness to academic performance but highlight the need for context-specific evidence, particularly in non-Western populations. Moreover, contextual factors may influence these relationships. Cultural norms, environmental conditions, and educational systems differ across regions, and findings from Western populations may not directly generalize to Middle Eastern contexts such as Qatar.

Despite high levels of physical inactivity reported among Qatari youth (Alnaqbi et al., 2025; Hermassi et al., 2024b), limited research has simultaneously examined physical activity patterns, sedentary behavior, health-related fitness, and academic achievement within this population. Examining these associations in Qatar is particularly relevant given climatic constraints on outdoor activity, sociocultural influences on sports participation, and evolving public health priorities. To our knowledge, no study in Qatar has simultaneously examined physical activity, sedentary behavior, and multidimensional fitness in relation to academic performance within a unified analytical framework.

The relationship between movement behaviors, fitness, and academic performance is complex and potentially confounded by multiple variables, including sex, biological maturation, anthropometric characteristics, socioeconomic status, sleep patterns, and nutritional factors. Biological maturation may influence physical activity participation, physical fitness performance, and academic achievement simultaneously and should therefore be considered a potential confounding factor when interpreting the observed associations. Sex differences in physical activity, fitness, and academic performance have been consistently reported in youth, with boys often exhibiting higher levels of vigorous activity and muscular strength, and girls sometimes showing superior balance or flexibility. Additionally, sex-related differences in academic outcomes, particularly in mathematics, have been observed in some contexts. In cross-sectional designs, these variables may influence both exposure (physical activity and fitness) and outcome (academic achievement), thereby limiting causal inference. Accounting for sex in the analyses, including the use of stratified comparisons, is therefore essential to clarify whether observed associations differ between boys and girls and to avoid confounding in interpreting physical activity–fitness–academic achievement relationships.

Given the relatively small sample size and the multifactorial nature of academic achievement, the present study does not attempt to establish directional or causal pathways. Instead, it adopts a comparative cross-sectional approach to examine concurrent differences between physically active and non-active schoolchildren. All findings should therefore be interpreted as associations rather than evidence of causation.

The present study aimed to examine differences between physically active and non-active Qatari schoolchildren in: (1) mathematics achievement; (2) health-related fitness components (strength, power, balance); (3) physical activity patterns and sedentary behavior; and (4) anthropometric and maturation indicators. The study explicitly considers sex as a factor in these analyses to examine potential sex-specific patterns and interactions, thereby providing a more nuanced understanding of how habitual physical activity and fitness relate to academic outcomes in boys and girls. Within this conceptual model, physical fitness may be considered a potential theoretical correlate in the relationship between physical activity and academic outcomes. However, the present study did not conduct a formal mediation analysis, and any mediating role of physical fitness should therefore be considered hypothetical. Qatari schoolchildren were selected as the population of interest because unique environmental, cultural, and educational factors may influence the relationships between physical activity, fitness, and academic achievement. Qatar's hot climate and limited opportunities for outdoor activity may constrain habitual physical activity compared with temperate regions, while sociocultural norms and gender-specific participation patterns may further shape engagement in organized sports and daily movement behaviors. Moreover, the rapid modernization of Qatar's educational system and evolving public health priorities create a context in which understanding these associations is particularly relevant for designing locally effective interventions. We hypothesized that physically active schoolchildren would demonstrate higher mathematics achievement, superior health-related fitness, more favorable activity patterns, and lower sedentary behavior than their non-active peers. However, consistent with the cross-sectional design, interpretations are restricted to associative relationships rather than causal inferences, which may inform future longitudinal or experimental investigations. These contextual and conceptual considerations underscore the importance of examining this population specifically, as patterns observed in Western populations may not generalize directly to Qatari youth.

This study addresses these gaps by examining the associations between physical activity, physical fitness, and academic achievement in Qatari schoolchildren, with explicit consideration of sex differences and contextual factors. By distinguishing between behavioral exposure and physiological adaptation, the study provides a framework for examining how physical activity, physical fitness, sedentary behavior, and mathematics achievement are associated in school-aged children.

## 2 Methods

### 2.1 Study design and participants

This cross-sectional study involved 126 schoolchildren (mean age: 12.2 ± 0.4 years) recruited from a single school in Doha, Qatar. The sample was selected using a convenience sampling approach, which may introduce selection bias. While recruitment from a single school allowed for standardized testing procedures, it limits the generalizability of the findings to other schools, regions, or populations with different demographic, cultural, or educational characteristics. Participants were divided into two groups: physically active (*n* = 63) and non-active (*n* = 63) based on their habitual physical activity levels. Physical activity was assessed using the International Physical Activity Questionnaire–Short Form (IPAQ-SF; Bhargava, 2017; Lee et al., 2011; Rääsk et al., 2017; Sember et al., 2020). Participants reporting ≥300 min/week of moderate-to-vigorous physical activity (MVPA) were classified as physically active, whereas those reporting <300 min/week were classified as non-active. Participation in organized sports was recorded for descriptive purposes only and was not used as a criterion for group allocation. The ≥300 min/week threshold was selected to reflect a practical, evidence-based cutoff for habitual activity in this age group, accounting for potential underreporting in self-administered questionnaires and the exclusion of weekend-specific activity in IPAQ-SF recall. While international guidelines recommend an average of 60 min per day (≈420 min per week), the ≥300 min/week threshold was selected as a pragmatic and previously used cutoff in questionnaire-based pediatric research. Furthermore, the same IPAQ-SF classification approach has been applied consistently in previous studies involving Qatari schoolchildren, thereby ensuring methodological consistency and facilitating comparisons across studies conducted within the same population and sociocultural context (Hermassi et al., 2024a,b, 2025).

Sample size estimation was performed using G^*^Power (version 3.1.2; Heinrich Heine University, Dusseldorf, Germany). Based on an anticipated effect size of η_p_^2^ = 0.14 [large; (Hopkins et al., 2009)], α = 0.05, power (1–β) = 0.80, and two groups, a minimum total sample size of 94 participants was required (Faul et al., 2007). The sample size calculation was performed to detect overall group differences and was not specifically powered to detect group × sex interaction effects across all study outcomes. Therefore, although the final sample size exceeded the minimum required sample size, the study may have had limited statistical power to detect small interaction effects.

Ethical approval for the study was obtained from the Institutional Review Board of Qatar University (QU-IRB 1544-FBA/21; initial approval on 20 May 2021 and renewal on 16 May 2024) and from the Ministry of Education and Higher Education of Qatar (REF: 18/2021). All procedures adhered to the principles of the Declaration of Helsinki. The study's objectives and procedures were explained to school administrators and physical education teachers. Prior to participation, parents or legal guardians provided written informed consent, and children provided written assent after receiving age-appropriate explanations about the study.

All physical fitness and academic assessments were conducted under consistent conditions, with trained personnel and calibrated equipment. Any deviations or adaptations made to accommodate the local school context were documented to ensure transparency and reliability of the data.

### 2.2 Inclusion and exclusion criteria

Children were eligible to participate if they met all of the following criteria: providing written informed consent from both themselves and a parent or legal guardian, being generally healthy with no medical conditions or restrictions that would limit participation in physical activity, having no physical impairments or disabilities that could interfere with exercise performance, and being aged between 10 and 13 years. Participants were excluded if they had a diagnosed psychological or neurodevelopmental disorder, including depression, anxiety, or attention deficit hyperactivity disorder (ADHD); were currently using medications known to affect mood, cognition, or the nervous system; lacked signed consent from a parent or legal guardian; or had irregular attendance or non-participation in scheduled physical education classes.

### 2.3 Experimental protocol

A cross-sectional design was used, with participants conveniently recruited from a single urban school in Doha, Qatar. Testing was conducted during regular physical education classes. Children were classified as physically active or non-active based on their self-reported moderate-to-vigorous physical activity (MVPA) levels obtained from the IPAQ-SF. Participants reporting ≥300 min/week of MVPA were classified as physically active, whereas those reporting <300 min/week were classified as non-active. Participation in organized sports was recorded for descriptive purposes only and was not used as a criterion for group allocation (Hermassi et al., 2021, 2023a, 2024c).

Data collection was completed over four sessions (08:00–10:00) under controlled outdoor conditions (24.5 ± 0.5 C; 65 ± 5% humidity). Participants were instructed to avoid vigorous exercise and ergogenic substances for 24 h prior to testing while maintaining their usual diet. Anthropometrics were assessed on day 1, the Stork balance test on day 2, and the medicine ball throw on day 3. The physical activity questionnaire was completed on day 4.

### 2.4 Variables and measuring instruments

#### 2.4.1 Anthropometry

Body mass was measured to the nearest 0.1 kg using a digital scale (Tanita TBF 105; Tanita Corporation of America, Inc., Arlington Heights, IL, USA). Standing height was assessed with a wall-mounted stadiometer to the nearest 0.5 cm. Arm span was measured with participants standing against a wall, arms extended horizontally, and the distance between the tips of the middle fingers was recorded to the nearest 0.5 cm. Body mass index (BMI) was then calculated using the standard formula:


$$\begin{array}{l} BMI\left(kg/m^{2}\right)=BodyMass\left(kg\right)÷Height^{2}\;\left(m\right) \end{array}$$

Arm span was included as a general anthropometric indicator of somatic growth and physical development. While it is not hypothesized to directly influence academic achievement, it provides context for interpreting variations in physical fitness and maturation.

#### 2.4.2 Biological maturity

The following two gender-specific equations were applied (Moore et al., 2015) to predict participants' maturity offset: Boys: Maturity offset (years) = −8.128741 + [0.0070346 (age sitting height)], Girls: Maturity offset (years) = −7.709133 + [0.0042232 (age stature)]. Maturity offset was estimated to characterize the biological maturity status of participants and to aid interpretation of physical performance outcomes. However, maturity offset was not included as a covariate in the statistical analyses because the primary aim of the study was to compare physically active and non-active children using a comparative cross-sectional approach rather than to perform multivariable analyses adjusting for potential confounding variables.

#### 2.4.3 Motor skills assessment

Participants' physical performance was assessed using three tests targeting distinct components of motor skills and health-related physical fitness:

- Stork balance test (motor skill)—participants stood on one leg with hands on hips and maintained balance for as long as possible. The best time from three trials was recorded (Miller et al., 2002). This test assesses postural control and static balance, a fundamental motor skill relevant to daily and sports activities.
- Medicine ball throw (muscular fitness)—Participants performed three overhead throws of a 3 kg ball, with the farthest distance recorded (Negrete et al., 2010). This test measures upper-body muscular power, an essential component of health-related fitness.
- Hand grip strength test (muscular fitness)—Dominant-hand strength was measured using a digital dynamometer (T.K.K. 5401, Tokyo, Japan) with 10 N sensitivity. This test evaluates maximal isometric muscular strength, a widely used indicator of overall muscular fitness in children.

These assessments were chosen because they are age-appropriate, feasible for large groups, and provide meaningful indicators of both fundamental motor skills (balance) and essential components of health-related physical fitness (strength and power). This distinction ensures clarity in interpreting the results, with balance reflecting a motor skill domain and the other tests reflecting fitness capacities relevant to health and academic performance (García-Hermoso et al., 2018; Hermassi et al., 2024b; Ortega et al., 2008).

#### 2.4.4 Academic performance

Academic performance was evaluated using students' mathematics percentage scores (%) obtained from the official school records for the 2021–2022 academic year. The mathematics percentage score was derived from the weighted combination of oral examinations, written examinations, and the final comprehensive examination according to the school's assessment policy. Physical activity, physical fitness, and anthropometric assessments were collected during the same academic term to ensure temporal alignment between the behavioral, physiological, and academic measures. Mathematics was selected as the academic outcome because it is a core subject that relies heavily on executive cognitive functions, including attention, working memory, and problem-solving, and has been widely used as an indicator of academic achievement in studies examining the relationship between physical activity, physical fitness, and educational outcomes (de Bruijn et al., 2018; Muntaner-Mas et al., 2022).

#### 2.4.5 Physical activity questionnaire

Physical activity was assessed using the International Physical Activity Questionnaire–Short Form (IPAQ-SF) (Mahfoudi et al., 2026), a widely validated instrument for estimating self-reported physical activity across diverse populations, including Middle Eastern countries such as Qatar (Craig et al., 2003). Although widely used, the IPAQ-SF relies on self-report and may overestimate physical activity compared with objective measures (e.g., accelerometry). The IPAQ-SF assesses activity across three intensity domains—vigorous (8 METs), moderate (4 METs), and walking (3.3 METs)—and calculates total physical activity in MET-minutes per week. Daily sitting time was also recorded to quantify sedentary behavior, an important indicator in pediatric health research (Lee et al., 2011).

Participants were classified as physically active if they reported ≥300 min of moderate-to-vigorous physical activity (MVPA) per week and as non-active if they reported <300 min per week. This threshold was selected as a pragmatic cutoff reflecting habitual activity while accounting for potential underreporting in younger populations and has been used previously in questionnaire-based epidemiological studies involving children and adolescents. Furthermore, the same classification approach has been applied consistently in previous studies involving Qatari schoolchildren, facilitating methodological comparability across investigations (Hermassi et al., 2023b, 2024a,b, 2025). Although international guidelines recommend approximately 60 min of MVPA per day (≈420 min/week), the present threshold was used to distinguish children reporting higher habitual activity levels from their less active peers. Therefore, the ≥300 min/week threshold should not be interpreted as equivalent to compliance with international physical activity guidelines, but rather as a pragmatic classification criterion used to distinguish relatively more active from less active children within the study sample.

The IPAQ-SF was administered to Qatari schoolchildren representing varied ages and body mass categories, consistent with prior regional applications (Hermassi et al., 2024a, 2025). The IPAQ-SF was self-administered by the participants during school hours under the supervision of trained researchers and physical education teachers. Standardized instructions were provided before questionnaire completion to ensure that all participants understood the questions and response options. No parental assistance was provided during questionnaire completion. The Arabic version of the IPAQ-SF was used, as it is the participants' native language. To enhance reliability, standardized instructions with culturally adapted examples relevant to the school context were provided to improve recall accuracy and consistent interpretation of activity types. Although the IPAQ-SF demonstrates acceptable psychometric properties, self-reported measures may overestimate activity compared with objective assessments such as accelerometry (Hsu et al., 2021).

Including sedentary time as a separate metric reflects growing evidence linking prolonged inactivity with adverse cognitive and metabolic outcomes, particularly relevant in Qatar, where childhood obesity and sedentary behaviors are increasingly prevalent. Collectively, the IPAQ-SF provided a comprehensive profile of habitual movement behaviors to examine associations with physical fitness and academic achievement.

### 2.5 Statistical analysis

Normality of data distribution was assessed using the Shapiro–Wilk test. To compare mathematics achievement, health-related fitness, physical activity patterns, and sedentary behavior between physically active and non-active Qatari schoolchildren, a two-way ANOVA [2 × 2; physical activity group (active vs. non-active) × sex (boys vs. girls)] was conducted for each outcome variable. Sex was included as a factor because of the well-established differences in physical activity patterns, fitness characteristics, and academic performance between boys and girls, thereby allowing the evaluation of both main effects and group × sex interactions. *Post-hoc* testing using Holm-Bonferroni probabilities adjustment was used to locate significant differences. All data are presented as mean ± SD. The alpha level for statistical significance was set at *p* < 0.05.

Associations between physical activity, sedentary behavior, and mathematics percentage scores were examined using Pearson's correlation coefficients. Correlation magnitudes were interpreted as follows: ≤ 0.29 = trivial, 0.30–0.49 = moderate, 0.50–0.69 = strong, 0.70–0.89 = very strong, and ≥ 0.90 = nearly perfect (Hopkins et al., 2009). Effect sizes were calculated using Cohen's *d* for pairwise comparisons, with values of 0.20–0.49 considered small, 0.50–0.79 medium, and ≥ 0.80 large (Cohen, 1988). Statistical analyses were performed using JASP version 19.1 (University of Amsterdam, Amsterdam, the Netherlands). No multivariable regression analyses were performed; therefore, the present analyses were limited to the evaluation of group differences and bivariate associations and do not permit conclusions regarding relationships after adjustment for potential confounding variables. Accordingly, the findings should be interpreted as unadjusted associations rather than associations adjusted for potential confounding variables.

## 3 Results

A significant difference in body mass between the active and non-active groups was found (*F* = 4.2, *p* = 0.042, Cohen's *d* = 0.37, 95% CI for mean difference: 0.3–4.5 kg), while a nearly significant difference was observed for arm span (*F* = 3.87, *p* = 0.052, Cohen's *d* = 0.35, 95% CI: −0.1–3.2 cm). For the other anthropometric parameters, including height, BMI, seat height, and biological maturity, sex and interaction effects were not significant (*F* ≤ 3.03, *p* ≥ 0.084, Cohen's *d* ≤ 0.30). *Post hoc* comparison revealed that body mass and arm span of the active boys were significantly lower and shorter, respectively, than those of non-active boys (Table 1).

**Table 1:** Anthropometrics of active and non-active children, stratified by sex.

	Boys		Girls	
Parameter	Active	Non-active	Active	Non-active
Age (yrs.)	12.3 ± 0.8	12.0 ± 0.5	12.3 ± 0.7	12.2 ± 0.5
Body mass (kg)	47.2 ± 12.9^*^	53.8 ± 15.0^*^	50.6 ± 9.5	53.8 ± 15.7
Height (m)	1.56 ± 0.11	1.59 ± 0.09	1.56 ± 0.08	1.59 ± 0.10
Body mass index (kg/m^2^)	19.0 ± 3.6	21.1 ± 4.7	20.9 ± 4.7	21.2 ± 5.6
Seat height (m)	1.21 ± 0.16	1.20 ± 0.05	1.22 ± 0.04	1.23 ± 0.04
Arm span (m)	1.51 ± 0.11^*^	1.58 ± 0.10^*^	1.57 ± 0.10	1.58 ± 0.10
Maturity (yrs.)	−1.0 ± 0.9	−1.1 ± 0.5	−1.2 ± 1.0	−1.0 ± 0.6

^*^indicates a significant difference between active and non-active boys (*p* < 0.05).

Analysis of the mathematics percentage scores in mathematics revealed a significant group effect between active and non-active children (*F* = 96.8, *p* < 0.001, Cohen's *d* = 1.7), whereas no other significant effects were observed (*F* ≤ 1.10, *p* ≥ 0.281, Cohen's *d* ≤ 0.28). *Post hoc* comparison showed that the active boys and girls had both higher mathematics percentage scores compared with the non-active boys and girls (Figure 1).

**Figure 1:**
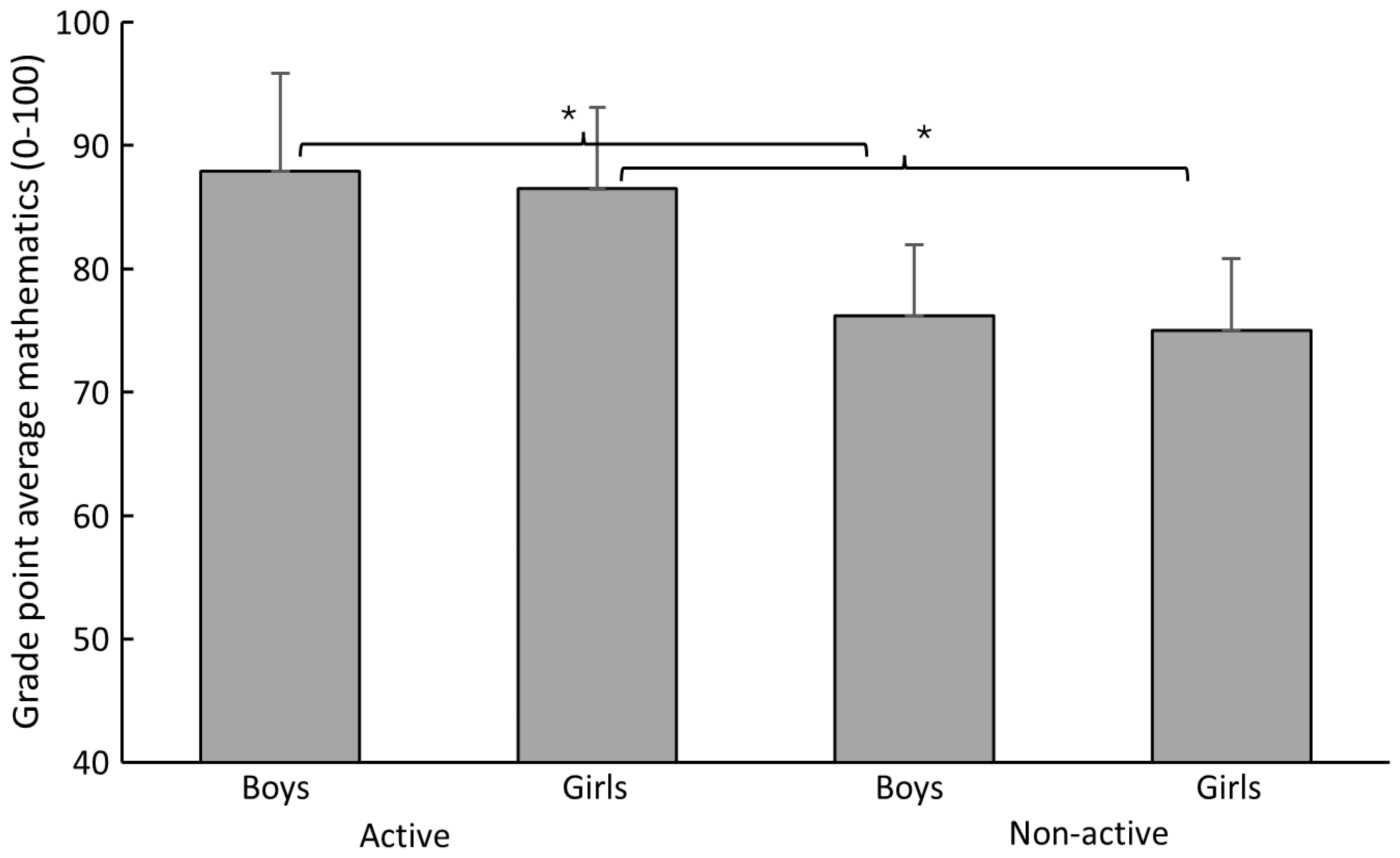
Mathematics percentage scores (%) of active and non-active boys and girls. ^*^Indicates a significant difference between active and non-active groups (*p* < 0.05).

The fitness tests showed a significant difference between the active and non-active groups for all tests (*F* ≥ 3.98, *p* ≤ 0.048, Cohen's *d* ≥ 0.36). A significant effect of sex was observed for the medicine ball throw and the handgrip strength test (*F* ≥ 4.01, *p* ≤ 0.047, Cohen's *d* ≥ 0.36), while no significant interaction effects were found (*F* ≤ 1.59, *p* ≥ 0.210, Cohen's *d* ≤ 0.26). *Post hoc* comparisons revealed that active boys threw farther than all other groups, and achieved longer durations in the Stork test compared to non-active boys and girls. In contrast, non-active boys exhibited significantly greater handgrip strength than active girls (Figure 2).

**Figure 2:**
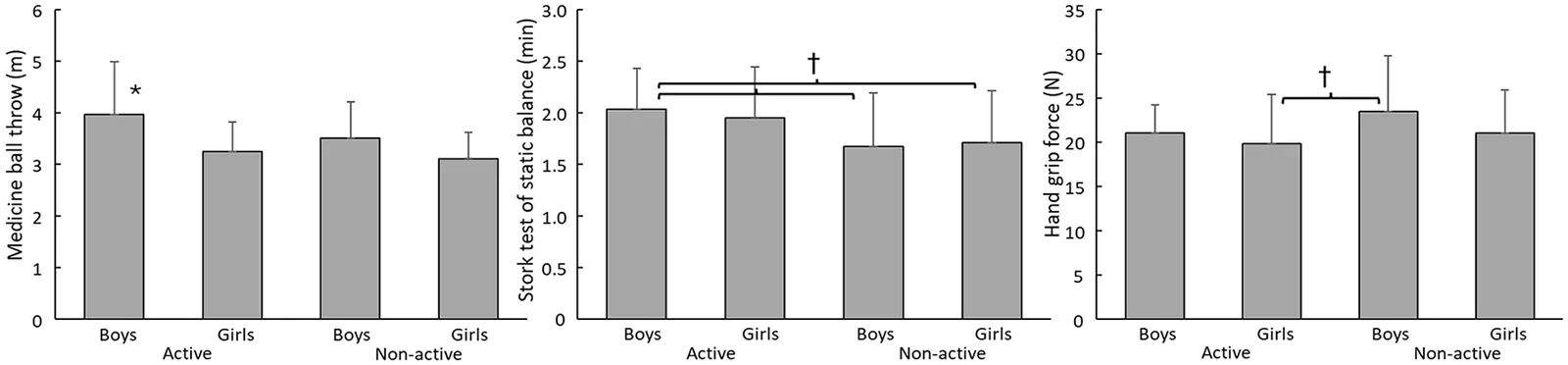
Physical fitness performance of active and non-active boys and girls for the medicine ball throw, Stork static balance test, and handgrip strength test. ^*^and ^†^ indicate significant differences between groups (*p* < 0.05).

Regarding reported physical activity levels and sedentary behavior, significant sex differences were found for vigorous activity (days, minutes and METS), sedentary behavior, and total MET per week (*F* ≥ 5.6, *p* ≤ 0.023, Cohen's *d* ≥ 0.41). In addition, a significant group effect was found for vigorous activity in METs per week (*F* = 4.06, *p* = 0.045, Cohen's *d* = 0.36) and for sedentary behavior (*F* = 31.3, *p* < 0.001, Cohen's *d* = 0.99). *Post hoc* comparison revealed that active boys reported in significantly higher levels of vigorous activity (all measured parameters) than non-active girls and also more vigorous activity than active girls when measured in METs per week. Furthermore, active boys exhibited significantly less sedentary behavior than the other three groups, while active girls engaged in less sedentary behavior than the non-active girls (Table 2).

**Table 2:** Physical activity level of active and non-active children, stratified by sex.

	Boys		Girls	
Activity level	Active	Non-active	Active	Non-active
Vigorous				
Days	2.9 ± 1.2^†^	2.4 ± 1.2	2.3 ± 1.1	2.0 ± 1.3^†^
Minutes per day	49.7 ± 16.3^†^	42.5 ± 18.9	39.7 ± 19.0	37.0 ± 19.7^†^
METs min/week	1,209 ± 732^‡^	904 ± 682	736 ± 520^‡^	597 ± 494^‡^
Moderate				
Days	2.8 ± 1.5	2.2 ± 1.1	2.5 ± 1.2	2.4 ± 1.4
Minutes per day	37.6 ± 17.1	37.5 ± 16.9	37.5 ± 15.7	28.7 ± 18.9
METs min/week	463 ± 381	358 ± 262	348 ± 227	262 ± 254
Walking				
Days	3.5 ± 1.7	3.2 ± 1.9	2.6 ± 1.9	3.2 ± 2.1
Minutes per day	39.4 ± 16.3	32.8 ± 20.2	32.0 ± 18.5	32.0 ± 17.5
METs min/week	502 ± 373	443 ± 474	323 ± 355	347 ± 336
Sedentary behavior (hrs.)	2.6 ± 1.3^*^	4.4 ± 1.7	3.7 ± 2.1^†^	5.3 ± 1.7^†^
Total METs per week (min)	2,175 ± 989	1,704 ± 1,046	1,407 ± 863	1,236 ± 884

^*^indicates a significant difference compared with all other groups (*p* < 0.05).

^†^indicates a significant difference between the active and non-active groups within the same sex (*p* < 0.05).

^‡^indicates a significant difference between active boys and both active girls and non-active girls (*p* < 0.05).

When evaluating the association between activity level, sedentary behavior, and mathematics percentage score, a significant moderate negative association was found between hours of sedentary behavior and mathematics percentage scores (*r* = −0.48, *p* < 0.001, 95% CI: −0.62 to −0.32), and a trivial positive correlation was observed between total METs per week and mathematics percentage scores (*r* = 0.18, *p* = 0.049, 95% CI: 0.001–0.35). No other significant correlations were found.

In summary, physically active schoolchildren demonstrated significantly lower body mass, higher mathematics percentage scores, and superior physical fitness performance than their non-active peers. Main effects of sex were observed for selected physical activity and fitness variables. Although active boys showed the highest descriptive values for vigorous activity and the lowest sedentary time, these observations should not be interpreted as evidence that the association between activity status and these outcomes differed by sex, given the absence of significant group × sex interactions. No significant differences were observed for height, BMI, seat height, or biological maturity. Furthermore, the present analyses did not assess the direct associations between individual physical fitness components and mathematics achievement.

## 4 Discussion

This study examined differences in physical activity patterns, health-related fitness, and mathematics achievement between physically active and non-active school-aged children in Qatar, with sex included as a factor. Overall, active children demonstrated lower body mass, higher mathematics percentage scores, and better performance across the physical fitness assessments than non-active children. Main effects of sex were also identified for selected physical activity and fitness outcomes. Although active boys displayed the highest descriptive vigorous activity levels and the lowest sedentary time, the absence of significant group × sex interactions indicates that the effect of activity status cannot be considered statistically different between boys and girls. Sedentary behavior showed a moderate negative association with mathematics percentage scores, whereas total weekly METs showed only a trivial positive correlation.

### 4.1 Anthropometry

Significant differences in body mass and arm span were observed between active and non-active boys, while height, BMI, seat height, and biological maturity did not differ meaningfully. The lower body mass in active boys aligns with prior research showing that regular physical activity is associated with healthier body composition in children (de Carvalho et al., 2024; Nowaczyk et al., 2023). Although arm span differences were only nearly significant, this measure provides context for somatic growth and body proportions, supporting interpretation of fitness outcomes rather than representing a direct determinant of academic performance. The absence of differences in other anthropometric measures may reflect the cross-sectional study design, the narrow age range (10–13 years), and the relatively small sample size, limiting observable effects on growth parameters.

Although a nearly significant difference in arm span was observed between active and non-active boys, this finding did not reach statistical significance and should therefore be interpreted with caution. Arm span was included primarily as an anthropometric indicator of somatic growth and body proportions. Given the cross-sectional design and lack of statistical significance, no conclusions can be drawn regarding the influence of physical activity on growth-related characteristics.

The lack of significant differences in height, BMI, seat height, and biological maturity between active and non-active children may be attributed to several factors (Harris et al., 2009). Firstly, the cross-sectional nature of the study limits the ability to infer causality. Longitudinal studies with extended follow-up periods are necessary to comprehensively understand the long-term effects of physical activity on various anthropometric measures. Secondly, the specific age group examined (10–13 years) may be at a stage where physical activity has yet to manifest noticeable effects on these parameters.

### 4.2 Physical activity

Sex-specific differences in vigorous activity, sedentary behavior, and total weekly METs were evident, with boys engaging in higher-intensity activity than girls, consistent with previous studies in Qatar and globally (Carson et al., 2016; Tremblay et al., 2011). Active boys accumulated more vigorous activity than both non-active girls and active girls, and spent less time sedentary than all other groups. Active girls also exhibited lower sedentary time than non-active girls. Moderate activity and walking did not differ significantly, suggesting that vigorous-intensity activity, rather than lower-intensity movement, was more strongly associated with differences in overall energy expenditure and associated fitness outcomes (Brazo-Sayavera et al., 2021; Ekelund et al., 2012). These findings are consistent with the potential value of structured, high-intensity activities within school and extracurricular programs to reduce sedentary behavior and promote equitable participation across sexes. Prior work in Qatar corroborates these patterns, highlighting both cultural and environmental factors that influence activity behavior (Hermassi et al., 2023b, 2024a,b, 2025).

The pronounced difference in sedentary behavior between active and non-active children aligns with prior evidence indicating that higher engagement in structured physical activity is associated with lower sedentary time (Biddle et al., 2004; Carson et al., 2016). Moreover, the greater MET values accumulated by active boys indicate that they are meeting recommended intensity thresholds for health benefits, which may partially explain the superior physical fitness and more favorable anthropometric characteristics reported in previous investigations (Strong et al., 2005). Interestingly, moderate-intensity activity and walking did not differ significantly between groups, suggesting that group differences were primarily driven by vigorous-intensity activity rather than routine lower-intensity movement. This finding underscores the potential value of interventions emphasizing vigorous physical activity to meaningfully increase energy expenditure and reduce sedentary time in youth populations (Ekelund et al., 2012).

Main effects of sex were observed for vigorous activity, sedentary behavior, and total weekly METs, while main effects of activity group were identified for vigorous activity expressed as MET-min/week and for sedentary behavior. The *post hoc* comparisons indicated that active boys reported higher vigorous activity than some of the other subgroups and lower sedentary time than the other groups. However, these subgroup comparisons should be interpreted cautiously and do not demonstrate that the association between activity status and these outcomes differed between boys and girls. Accordingly, the findings support separate contributions of activity status and sex to selected movement behaviors rather than a sex-dependent effect of activity status.

The observed main effect of sex is consistent with previous research reporting differences in physical activity patterns between boys and girls in Qatari and international youth populations (Hermassi et al., 2023b, 2024a,b, 2025). Similarly, the main effect of activity group supports an association between activity classification and vigorous activity or sedentary behavior, which is in agreement with previous findings in school-aged children (Hermassi et al., 2023b, 2024a,b). Nevertheless, because group × sex interactions were not statistically significant, the present findings do not provide sufficient evidence that being classified as active was associated with a greater benefit in boys than in girls. Future studies with larger samples and adequate statistical power to examine interaction effects are needed to determine whether activity-related differences genuinely vary by sex.

Incorporating structured, high-intensity activities into school curricula and after-school programs may help reduce sedentary time and promote equitable physical activity participation across sexes. Future longitudinal studies are warranted to explore the temporal relationships and potential causal pathways between sex, activity status, sedentary behavior, and long-term health outcomes.

### 4.3 Physical fitness

Activity status significantly influenced all fitness measures, indicating generally better fitness performance among active than non-active children. Significant main effects of sex were observed for the medicine ball throw and handgrip strength tests. However, no significant group × sex interactions were observed. Therefore, although active boys demonstrated the highest descriptive values for some fitness outcomes, these findings do not indicate that activity status had a greater effect in boys than in girls. These findings are consistent with previous evidence indicating that regular physical activity is associated with improved muscular strength, power, and neuromotor fitness in youth (Badau et al., 2021; Chen et al., 2018; Guo et al., 2022; Hermassi et al., 2023a, 2024b, 2025; Mateo-Orcajada et al., 2022).

Our findings are also consistent with prior research conducted in Qatar (Hermassi et al., 2023a, 2024b, 2025), which has shown that children engaged in regular physical activity achieve higher scores in health-related fitness components, including muscular strength, power, and balance, compared with less active peers (Faul et al., 2007; Hermassi et al., 2024b). Moreover, the observed sex differences mirror those reported by Hermassi et al. (2023a), where boys outperformed girls in upper-body strength and explosive power, while balance differences were comparatively smaller.

Additional international evidence supports these findings. Mateo-Orcajada et al. (2022) demonstrated that active adolescents outperform sedentary peers in multiple fitness domains. Similarly, Guo et al. (2022) reported that higher physical fitness levels were associated with superior cardiorespiratory and overall fitness profiles in female college students. Chen et al. (2018) found that elementary school children engaging in greater physical activity performed better on standardized fitness assessments (e.g., PACER, curl-ups, push-ups, and trunk lift) than less active peers. Furthermore, Badau et al. (2021) showed that physically active students exhibited greater muscle mass compared with semi-active counterparts. Together, these findings converge with our results, underscoring the robust association between regular physical activity and multidimensional fitness development in school-aged populations.

These findings are consistent with current recommendations promoting structured and vigorous physical activity within school curricula to optimize muscular strength, power, balance, and overall physical development. They also emphasize the need for gender-responsive physical education strategies to ensure equitable opportunities for girls to develop strength and power capacities, thereby reducing performance disparities.

### 4.4 Academic school performance

Active children, regardless of sex, achieved higher mathematics percentage scores than their non-active peers. This finding is consistent with previous studies reporting associations between habitual physical activity, physical fitness, executive function, attention, and problem-solving abilities, all of which are related to academic performance (Alfaro-Chaverri et al., 2025; Castelli et al., 2007; Donnelly et al., 2016; Hermassi et al., 2024b; Van Dusen et al., 2011; Zhang et al., 2025). Sedentary behavior showed a moderate negative association with mathematics percentage scores, underscoring the potential benefits of reducing inactivity in school-aged populations. Collectively, these findings indicate that physically active children demonstrated both higher physical fitness and better mathematics achievement. However, because direct associations between individual physical fitness measures and mathematics achievement were not examined, the contribution of specific fitness components cannot be determined.

Although previous studies have suggested that regular physical activity may be associated with favorable neurocognitive processes, including executive function, attention, and working memory, these variables were not directly assessed in the present study. Therefore, any reference to neurophysiological mechanisms, such as changes in cerebral blood flow or neuroplasticity, should be considered hypothetical and based on previous literature rather than on the findings of the present study. Consequently, the observed associations between physical activity, physical fitness, sedentary behavior, and mathematics achievement should not be interpreted as evidence of specific underlying biological or cognitive mechanisms. Future longitudinal and experimental studies incorporating direct assessments of neurocognitive function and neurophysiological outcomes are required to clarify these potential mechanisms.

Prior research in Qatar reinforces these findings. Hermassi et al. (2024b) reported that physically active children outperformed less active peers in mathematics achievement tests, highlighting the value of structured physical activity programs. Similarly, Zhang et al. (2025) observed positive associations between frequent vigorous physical activity and mathematics performance, while Alfaro-Chaverri et al. (2025) found that habitual activity correlated with higher standardized test scores, including in mathematics. These studies collectively suggest that regular physical activity may be associated with cognitive function, concentration, and problem-solving skills, although these variables were not directly assessed in the present study.

Additional international studies provide further support: Donnelly et al. (2016) demonstrated improvements in academic performance, particularly mathematics, following daily physical activity interventions. Van Dusen et al. (2011) found that higher cardiorespiratory fitness was associated with better standardized test scores in math and reading among U.S. adolescents. Castelli et al. (2007) observed that higher fitness levels positively influenced cognitive performance and classroom behavior, indirectly enhancing academic outcomes. Taken together, this body of evidence highlights the potential role of regular physical activity and fitness in supporting both cognitive and academic development in school-aged children.

This study has several limitations. Primarily, the cross-sectional design precludes causal conclusions regarding the relationships among physical activity, anthropometry, fitness, and academic outcomes. Second, self-reported physical activity and sedentary behavior may be affected by recall bias. Third, focusing on a single age group limits generalizability across developmental stages. Finally, unmeasured confounders such as nutrition, sleep, and socioeconomic status may have influenced the observed associations. Despite these limitations, the findings provide valuable insights into the positive links between physical activity, fitness, and academic achievement in Qatari schoolchildren.

### 4.5 Practical implications for schools and policy

The findings of this study demonstrate an association between physical activity, physical fitness, sedentary behavior, and academic achievement among school-aged children. Although the cross-sectional design does not permit causal inference, these findings are consistent with current public health recommendations that encourage regular physical activity as part of a healthy lifestyle. The following practical considerations are therefore informed by existing public health evidence rather than by causal evidence from the present study. Schools may consider providing regular opportunities for moderate-to-vigorous physical activity, incorporating active learning strategies into classroom instruction, and reducing prolonged sedentary time to support children's overall health and wellbeing.

Moreover, inclusive and gender-sensitive physical education programs may help reduce disparities in activity levels between boys and girls, ensuring equitable opportunities for participation in physical activity. At the policy level, collaboration between education and health authorities may support the implementation of evidence-based school physical activity initiatives. However, the present findings should not be interpreted as evidence that increasing physical activity will directly improve academic achievement. Establishing such causal relationships requires well-designed longitudinal and intervention studies.

### 4.6 Limitations

This study has several limitations that should be considered. First, the relatively small sample size may have limited the statistical power to detect smaller associations and group × sex interaction effects. Therefore, findings concerning non-significant associations and interaction effects should be interpreted with caution. Second, physical activity and sedentary behavior were assessed using self-reported data and may therefore be affected by recall bias, social desirability bias, and inaccurate estimation of the frequency, duration, and intensity of physical activity. These limitations may have introduced measurement error and misclassification of participants' activity levels. Furthermore, although the IPAQ-SF has been used in pediatric populations, children may experience difficulty accurately recalling their habitual physical activity. In addition, although the Arabic version of the IPAQ has been used in Arabic-speaking populations, recent evidence indicates that psychometric evaluation of Arabic physical activity questionnaires remains incomplete, and validation evidence specific to Arabic-speaking children aged 10–13 years is limited (Mahfoudi et al., 2026). Therefore, measurement error related to self-reported physical activity cannot be excluded and should be considered when interpreting the findings. Future studies should use larger, more representative samples and incorporate objective measures, such as accelerometry, to improve the accuracy of physical activity and sedentary behavior assessment.

Third, the present study was conducted in a single urban school in Doha, which allowed for standardized procedures but limits the generalizability of findings to the broader population of Qatari schoolchildren. Although recruiting participants from a single school reduced variability in school policies, curriculum, and assessment procedures, it also limited variability in socioeconomic and educational environments, which may influence the observed associations. Future research should aim to recruit participants from multiple schools across diverse regions and socioeconomic backgrounds to improve external validity and ensure that results are nationally representative.

Fourth, unmeasured confounders, including nutrition, sleep, socioeconomic status, and classroom environment, may have influenced the observed outcomes. The use of mathematics percentage score alone as an indicator of academic achievement may not fully capture overall academic performance across subjects. Furthermore, sedentary behavior was assessed only as total daily sitting time using the IPAQ-SF. Consequently, the present study could not distinguish between different sedentary activities such as classroom sitting, homework, reading, screen time, gaming, or other sedentary behaviors, which may have different associations with mathematics achievement. Specifically, we did not collect data on potential confounding variables such as nutritional status, sleep quality, socioeconomic status, parental education, parental support, prior academic achievement, private tutoring, motivation, or teacher/classroom factors, all of which may influence mathematics achievement. Furthermore, prior academic achievement or baseline mathematics performance was not assessed. Consequently, it is unclear whether the active and non-active groups differed academically before the current assessment period.

Furthermore, although biological maturity was estimated using maturity offset, it was not included as a covariate in the statistical analyses. Consequently, the potential influence of biological maturation on physical activity, physical fitness, and mathematics achievement could not be statistically controlled and may have confounded the observed associations. Future studies should incorporate biological maturity as a covariate in multivariable analytical models to better account for its potential confounding effects.

Fifth, cardiorespiratory fitness was not assessed in the present study. Given that cardiorespiratory fitness has consistently been associated with cognitive function and academic achievement in children and adolescents, the absence of an aerobic fitness measure limits direct comparison with previous studies and may have restricted a more comprehensive evaluation of the relationships between physical fitness and mathematics achievement. Future studies should incorporate objective assessments of cardiorespiratory fitness to better clarify its association with academic performance.

Sixth, although Holm-Bonferroni adjustments were applied for *post hoc* comparisons, multiple outcome variables and correlation analyses were examined. Consequently, the possibility of Type I error cannot be completely excluded, and the findings should be interpreted with caution.

Despite these limitations, the present study provides preliminary insights into the associations between habitual physical activity, physical fitness, and academic achievement among school-aged children in Qatar. These findings contribute to the growing evidence on the associations between regular physical activity, particularly vigorous-intensity activity, and health-related and academic outcomes in school-aged children, while highlighting the importance of further investigating potential sex-related patterns in future longitudinal and intervention studies.

## 5 Conclusions

Physically active schoolchildren showed lower body mass, higher mathematics achievement, and superior performance in health-related fitness tests, including balance, muscular strength, and explosive power, compared with their non-active peers. Main effects of activity status and sex were observed for several physical activity and fitness outcomes. Although active boys demonstrated the highest descriptive values for vigorous activity and certain fitness measures, the absence of significant group × sex interactions indicates that activity-related differences did not statistically differ between boys and girls.

These findings suggest that habitual physical activity, particularly when accompanied by lower sedentary behavior and higher physical fitness, is associated with favorable physiological and academic outcomes. Because of the cross-sectional design, these findings should be interpreted as associations only and do not establish causal relationships. However, the trivial correlation observed between total physical activity volume (total METs/week) and mathematics achievement indicates that overall activity volume alone is unlikely to explain academic performance. Physically active children demonstrated both higher physical fitness and better mathematics achievement than their non-active peers. However, the present analyses did not directly examine the associations between individual physical fitness components and mathematics achievement. Therefore, no conclusions can be drawn regarding the contribution of specific fitness measures to academic performance. Importantly, these findings are consistent with the potential role of structured physical education in supporting children's physical fitness and reducing sedentary behavior. By encouraging participation in structured, vigorous physical activities, schools and families may contribute to reducing sedentary behavior, which may in turn be associated with favorable physical and cognitive outcomes while helping children establish lifelong healthy habits. Furthermore, ensuring equitable opportunities for participation in physical activity among both boys and girls may help support physical fitness and learning outcomes.

However, these conclusions should be interpreted in light of the study's limitations, including its cross-sectional design, reliance on self-reported activity data, and the single-school sample, which may limit generalizability. Future research should employ longitudinal or intervention-based designs, incorporate objective measures of physical activity, and recruit from multiple schools to confirm and extend these findings. These insights can inform educational policy and the design of school-based physical activity programs, supporting the integration of regular, structured, and inclusive physical education as a core component of holistic child development. By fostering environments that prioritize physical fitness and reduce sedentary behavior, educators and policymakers can contribute not only to the physical health of children but also to their academic and lifelong success.

## Data Availability

The datasets generated and/or analyzed during the current study are available from the corresponding authors upon reasonable request.

## References

[B1] Alfaro-Chaverri A. Miller A. Noonan C. Quindry J. Gaskill S. E. (2025). A cross-sectional study of 2nd–12th grade students' physical activity related to academic achievement. Sports Med. Health Sci. 8, 537–548. doi: 10.1016/j.smhs.2025.03.011 42539936 PMC13424724

[B2] Alnaqbi S. E. Sohail R. Radwan H. M. Mohamad M. N. Zeb F. Hasan H. . (2025). Physical activity, screen time, diet and health outcomes in children in MENA region: a narrative review. Front. Public Health. 13:1628904. doi: 10.3389/fpubh.2025.1628904 41069803 PMC12504483

[B3] Aly M. Hassan MD. Hassan MM. Alibrahim M. Kamijo K. (2024). Association of aerobic fitness and grip strength with cognitive and academic performance in Arab children. Prog. Brain Res. 286, 107–128. doi: 10.1016/bs.pbr.2023.11.005 38876572

[B4] Badau D. Badau A. Trambitas C. Trambitas-Miron D. Moraru R. Stan A. A. . (2021). Differences in body composition in active vs semi-active students. IJERPH. 18:7906. doi: 10.3390/ijerph18157906 34360200 PMC8345541

[B5] Barbosa A. Whiting S. Simmonds P. Scotini Moreno R. Mendes R. Breda J. (2020). Physical activity and academic achievement: an umbrella review. Int. J. Environ. Res. Public Health. 17:5972. doi: 10.3390/ijerph17165972 32824593 PMC7460146

[B6] Bertrand C. Steinberg L. Duell N. Di Giunta L. Dodge K. A. Gurdal S. . (2025). Physical activity and two-year change in adolescent well-being in nine countries. J. Res. Adolesc. 35:e70035. doi: 10.1111/jora.70035 40411243 PMC12232549

[B7] Bhargava M. (2017). Use of IPAQ-SF for assessing physical activity in children. J. Fam. Commun. Med. 24:131. doi: 10.4103/jfcm.JFCM_60_16 28566980 PMC5426106

[B8] Biddle S. J. Gorely T. Stensel D. J. (2004). Health-enhancing physical activity and sedentary behaviour in youth. J. Sports Sci. 22, 679–701. doi: 10.1080/02640410410001712412 15370482

[B9] Bielik V. Nosál V. Nechalová L. Špánik M. Žilková K. Grendar M. (2025). Prediction model of academic achievement based on cardiorespiratory fitness and BMI in ninth-grade students. BMC Pediatr. 25:20. doi: 10.1186/s12887-024-05353-2 39789497 PMC11715446

[B10] Brazo-Sayavera J. Aubert S. Barnes J. D. González S. A. Tremblay M. S. (2021). Gender differences in PA and sedentary behavior in Latin-American youth. PLoS One. 16:e0255353. doi: 10.1371/journal.pone.0255353 34383803 PMC8360534

[B11] Carson V. Hunter S. Kuzik N. Gray C. E. Poitras V. J. Chaput J. P. . (2016). Sedentary behaviour and health indicators in youth: update. Appl. Physiol. Nutrit. Metabol. 41, S240–S265. doi: 10.1139/apnm-2015-0630 27306432

[B12] Casanova F. O'Loughlin J. Karageorgiou V. Beaumont R. N. Bowden J. Wood A. R. . (2023). Effects of physical activity and sedentary time on depression, anxiety and well-being: a bidirectional Mendelian randomisation study. BMC Med. 21:501. doi: 10.1186/s12916-023-03211-z 38110912 PMC10729457

[B13] Caspersen C. J. Powell K. E. Christenson G. M. (1985). Physical activity, exercise, and physical fitness: definitions and distinctions for health-related research. Public Health Rep. 100, 126–131. 3920711 PMC1424733

[B14] Castelli D. M. Hillman C. H. Buck S. M. Erwin H. E. (2007). Fitness and academic achievement in elementary students. J. Sport Exer. Psychol. 29, 239–252. doi: 10.1123/jsep.29.2.239 17568069

[B15] Castillo-Navarrete J. L. Guzmán-Castillo A. Bustos C. (2024). Longitudinal analysis of academic stress and its effects on salivary biomarkers and academic outcomes: study protocol. PLoS One. 19:e0315650. doi: 10.1371/journal.pone.0315650 39705290 PMC11661652

[B16] Chen W. Hammond-Bennett A. Hypnar A. Mason S. (2018). Health-related fitness and PA in elementary students. BMC Public Health. 18:195. doi: 10.1186/s12889-018-5107-4 29378563 PMC5789625

[B17] Cohen J. (1988). Statistical Power Analysis for the Behavioral Sciences. Hillsdale, NJ: Lawrence Erlbaum Associates. doi: 10.4324/9780203771587

[B18] Craig C. L. Marshall A. L. Sjöström M. Bauman A. E. Booth M. L. Ainsworth B. E. . (2003). International physical activity questionnaire reliability and validity. Med. Sci. Sports Exerc. 35, 1381–1395. doi: 10.1249/01.MSS.0000078924.61453.FB 12900694

[B19] de Bruijn A. G. M. Hartman E. Kostons D. Visscher C. Bosker R. J. (2018). Physical fitness, executive functioning, and low academic achievement. J. Exp. Child Psychol. 167, 204–221. doi: 10.1016/j.jecp.2017.10.010 29179006

[B20] de Carvalho B. K. G. Ribeiro A. P. João S. M. A. (2024). Physical activity and anthropometry in youth. J. Manipulative Physiol. Ther. 47, 45–57. doi: 10.1016/j.jmpt.2024.08.013 39425705

[B21] Donnelly J. E. Hillman C. H. Castelli D. Etnier J. L. Lee S. Tomporowski P. . (2016). PA, fitness, cognition, and academic achievement: systematic review. MSSE. 48:1197. doi: 10.1249/MSS.0000000000000901 27182986 PMC4874515

[B22] Ekelund U. Luan J. Sherar L. B. Esliger D. W. Griew P. Cooper A. (2012). MVPA, sedentary time, and cardiometabolic risk in youth. JAMA. 307, 704–712. doi: 10.1001/jama.2012.156 22337681 PMC3793121

[B23] Faul F. Erdfelder E. Lang A. G. Buchner A. (2007). G^*^Power 3: a flexible power analysis program. Behav. Res. Methods. 39, 175–191. doi: 10.3758/BF03193146 17695343

[B24] García-Hermoso A. Ramírez-Vélez R. Ramírez-Campillo R. Peterson M. D. Martínez-Vizcaíno V. (2018). Aerobic + resistance vs aerobic alone for pediatric obesity: meta-analysis. Br. J. Sports Med. 52, 161–166. doi: 10.1136/bjsports-2016-096605 27986760

[B25] Guo M. M. Wang X. Z. Koh K. T. P. (2022). A sedentary time, and fitness in female college students. BMC Women's Health. 22:502. doi: 10.1186/s12905-022-02108-y 36476426 PMC9727854

[B26] Guthold R. Cowan M. J. Autenrieth C. S. Kann L. Riley L. M. (2010). Physical activity and sedentary behavior among schoolchildren: a 34-country comparison. J. Pediatr. 157, 43–49. doi: 10.1016/j.jpeds.2010.01.019 20304415

[B27] Harris K. C. Kuramoto L. K. Schulzer M. Retallack J. E. (2009). School-based PA interventions and BMI in children: meta-analysis. CMAJ. 180, 719–726. doi: 10.1503/cmaj.080966 19332753 PMC2659836

[B28] Hermassi S. Chelly M. S. Michalsik L. B. Sanal N. E. M. D Hayes L. Cadenas-Sanchez C. (2021). Fatness, fitness, and academic performance in handball players. PLoS One. 16:e0246476. doi: 10.1371/journal.pone.0246476 33606780 PMC7895367

[B29] Hermassi S. Dhahbi W. Jebabli N. Alneama A. Ketelhut S. van den Tillaar R. (2025). Difference in out-of-school physical activity, sedentary behavior, and fitness in relation to mathematics achievement: a cross-sectional study. Front. Educ. 10:1590300. doi: 10.3389/feduc.2025.1590300

[B30] Hermassi S. Hayes L. D. Bartels T. Schwesig R. (2023a). Differences in body composition, static balance, field test performance, and academic achievement in 10–12-year-old soccer players. Front. Physiol. 14:1150484. doi: 10.3389/fphys.2023.1150484 37064919 PMC10097943

[B31] Hermassi S. Ketelhut S. Konukman F. Ayari M. A. Al-Marri S. Al Rawahi N. . (2024a). Differences in activity, sedentary behavior, fitness and academic achievement in normal-weight and obese children in Qatar. J. Clin. Med. 13:1057. doi: 10.3390/jcm13041057 38398370 PMC10888728

[B32] Hermassi S. Ketelhut S. Konukman F. Sellami M. Al-Marri S. Nigg C. R. . (2024b). Comparative analysis of physical activity, fitness, and academic achievements in schoolchildren in Qatar. Healthcare. 12:588. doi: 10.3390/healthcare12050588 38470699 PMC10930824

[B33] Hermassi S. Konukman F. Al-Marri S. S. Hayes L. D. Bartels T. Schwesig R. (2024c). Biological maturation, performance, postural control, and math achievement in youth soccer players. PLoS One. 19:e0298301. doi: 10.1371/journal.pone.0298301 38451897 PMC10919613

[B34] Hermassi S. Konukman F. Hayes L. D. Schwesig R. (2023b). PE and gender differences in PA related to academic success. Appl. Sci. 13:10771. doi: 10.3390/app131910771

[B35] Hopkins W. G. Marshall S. W. Batterham A. M. Hanin J. (2009). Progressive statistics for studies in sports medicine and exercise science. Med. Sci. Sports Exerc. 41, 3–12. doi: 10.1249/MSS.0b013e31818cb278 19092709

[B36] Hsu Y. W. (2021). Subjective vs objective daily physical activity in college students. J. Sci. Sport Exerc. 3, 186–194. doi: 10.1007/s42978-019-00047-z

[B37] Hu D. Zhou S. Crowley-McHattan Z. J. Liu Z. (2024). A comparative study of the physical activity guidelines for children and adolescents from five countries and WHO. Front. Public Health. 12:1421843. doi: 10.3389/fpubh.2024.1421843 39071153 PMC11272551

[B38] Kao S. L. Wang J. H. Chen S. C. Li Y. Y. Yang Y. L. Lo R. Y. (2021). Impact of comorbidity burden on cognitive decline in older adults with dementia. Dement. Geriatr. Cogn. Disord. 50, 43–50. doi: 10.1159/000514651 33789290

[B39] Ketelhut S. Ketelhut K. Ketelhut S. R. Ketelhut R. G. (2024). Effects of school-based high-intensity interval training on hemodynamic parameters and heart rate variability. J. Strength Cond. Res. 38, 1033–1040. doi: 10.1519/JSC.0000000000004744 38349394

[B40] Ketelhut S. Ketelhut S. R. Ketelhut K. (2020). School-based exercise intervention improves blood pressure and parameters of arterial stiffness in children: a randomized controlled trial. Pediatr. Exerc. Sci. 33, 1–7. doi: 10.1123/pes.2020-0053 32937598

[B41] Lee P. H. Macfarlane D. J. Lam T. H. Stewart S. M. (2011). Validity of the IPAQ-SF: a systematic review. Int. J. Behav. Nutrit. Phys. Activ. 8:115. doi: 10.1186/1479-5868-8-115 22018588 PMC3214824

[B42] Li D. Wang D. Zou J. Li C. Qian H. Yan J. . (2023). Effect of physical activity interventions on children's academic performance: a meta-analysis. Eur. J. Pediatr. 182, 3587–3601. doi: 10.1007/s00431-023-05009-w 37227500

[B43] Mahfoudi H. Benmaamar S. Kirat M. El Harch I. Bahra N. Zahrae Bartal F. . (2026). Systematic review of physical activity measurement scales validated for Arabic-speaking populations: insights from the BRIDGE project. Front. Public Health. 14:1814479. doi: 10.3389/fpubh.2026.1814479 42040138 PMC13108486

[B44] Martin-Martinez C. Valenzuela P. L. Martinez-Zamora M. Martinez-de-Quel Ó. (2023). School-based physical activity interventions and language skills: a systematic review and meta-analysis. J. Sci. Med. Sport. 26, 140–148. doi: 10.1016/j.jsams.2022.12.007 36609085

[B45] Mateo-Orcajada A. González-Gálvez N. Abenza-Cano L. Vaquero-Cristóbal R. (2022). Differences in fitness and body composition between active and sedentary adolescents: meta-analysis. J. Youth Adolesc. 51, 177–192. doi: 10.1007/s10964-021-01552-7 35031910

[B46] Miller W. C. Speechley M. Deathe A. B. (2002). Balance confidence among people with lower-limb amputations. Phys. Ther. 82, 856–865. doi: 10.1093/ptj/82.9.856 12201800

[B47] Moore S. A. McKay H. A. Macdonald H. Nettlefold L. Baxter-Jones A. D. Cameron N. . (2015). Enhancing a somatic maturity prediction model. Med. Sci. Sports Exerc. 47, 1755–1764. doi: 10.1249/MSS.0000000000000588 25423445

[B48] Muntaner-Mas A. Mazzoli E. Abbott G. Mavilidi M. F. Galmes-Panades A. M. (2022). Do fitness and executive function mediate PA–achievement links? Children. 9:823. doi: 10.3390/children9060823 35740760 PMC9221993

[B49] Negrete R. J. Hanney W. J. Kolber M. J. Davies G. J. Ansley M. K. McBride A. B. . (2010). Reliability and normative values for tests of upper extremity function. J. Strength Cond. Res. 24, 3318–3325. doi: 10.1519/JSC.0b013e3181e7259c 21088548

[B50] Nowaczyk M. Cieślik K. Waszak M. (2023). Impact of increased physical activity on body mass in children. Children. 10:764. doi: 10.3390/children10050764 37238312 PMC10217524

[B51] Ortega F. B. Ruiz J. R. Castillo M. J. Sjöström M. (2008). Physical fitness in childhood and adolescence: a marker of health. Int. J. Obes. 32, 1–11. doi: 10.1038/sj.ijo.0803774 18043605

[B52] Rääsk T. Mäestu J. Lätt E. Jürimäe J. Jürimäe T. Vainik U. . (2017). Comparison of IPAQ-SF with accelerometer in adolescent boys. PLoS One. 12:e0169527. doi: 10.1371/journal.pone.0169527 28056080 PMC5215940

[B53] Robinson K. Riley N. Owen K. Drew R. Mavilidi MF. Hillman CH. . (2023). Effects of resistance training on academic outcomes in youth: a systematic review and meta-analysis. Sports Med. 53, 2095–2109. doi: 10.1007/s40279-023-01881-6 37466900 PMC10587249

[B54] Rodriguez-Ayllon M. Cadenas-Sánchez C. Estévez-López F. Muñoz N. E. Mora-Gonzalez J. Migueles J. H. . (2019). Role of physical activity and sedentary behavior in the mental health of preschoolers, children and adolescents: a systematic review and meta-analysis. Sports Med. 49, 1383–1410. doi: 10.1007/s40279-019-01099-5 30993594

[B55] Sember V. Jurak G. Kovač M. Morrison S. A. Starc G. (2020). Children's physical activity, academic performance, and cognition: a systematic review. Front. Public Health. 8:536635. doi: 10.3389/fpubh.2020.00307 32760689 PMC7372103

[B56] Shah S. S. Diwan S. J. (2025). Does physical fitness impact academic achievement in school children? Bullet. Facul. Phys. Therapy. 30:54. doi: 10.1186/s43161-025-00309-8

[B57] Singh B. Bennett H. Miatke A. Dumuid D. Curtis R. Ferguson T. . (2025). Effectiveness of exercise for improving cognition and executive function: an umbrella review. Br. J. Sports Med. 59, 866–876. doi: 10.1136/bjsports-2024-108589 40049759 PMC12229068

[B58] Spruit A. Assink M. van Vugt E. van der Put C. Stams G. J. J. M. (2016). The effects of physical activity interventions on psychosocial outcomes in adolescents: a meta-analytic review. Clin. Psychol. Rev. 45, 56–71. doi: 10.1016/j.cpr.2016.03.006 27064552

[B59] Strong W. B. Malina R. M. Blimkie C. J. Daniels S. R. Dishman R. K. Gutin B. . (2005). Evidence-based physical activity for school-age youth. J. Pediatr. 146, 732–737. doi: 10.1016/j.jpeds.2005.01.055 15973308

[B60] Teuber M. Leyhr D. Sudeck G. (2024). Physical activity improves stress load, recovery, and academic performance-related parameters among university students: a longitudinal study. BMC Public Health. 24:598. doi: 10.1186/s12889-024-18082-z 38402396 PMC10893600

[B61] Tremblay M. S. LeBlanc A. G. Kho M. E. Saunders T. J. Larouche R. Colley R. C. . (2011). Sedentary behaviour and health in youth: systematic review. IJBNPA. 8:98. doi: 10.1186/1479-5868-8-98 21936895 PMC3186735

[B62] Van Dusen D. P. Kelder S. H. Kohl H. W. III. Ranjit N. Perry C. L. (2011). Associations of fitness and academic performance. J. School Health. 81, 733–740. doi: 10.1111/j.1746-1561.2011.00652.x 22070504

[B63] Wang K. Li Y. Liu H. Zhang T. Luo J. (2024). Can physical activity counteract the negative effects of sedentary behavior on the physical and mental health of children and adolescents? A narrative review. Front. Public Health. 12:1412389. doi: 10.3389/fpubh.2024.1412389 39157520 PMC11328546

[B64] Zhang Z. (2025). A dual-process framework for how physical activity enhances academic performance via executive functions. Educ. Psychol. Rev. 37:68. doi: 10.1007/s10648-025-10049-9

